# Dosimetric and clinical outcomes of stereotactic body radiotherapy for primary lung cancer: isocenter-based vs. volume-based prescription

**DOI:** 10.3389/fmed.2026.1763203

**Published:** 2026-05-18

**Authors:** Mitsuru Okubo, Tomohiro Itonaga, Tatsuhiko Zama, Ryuji Mikami, Yukinori Okada, Tsubasa Kawamoto, Shiho Wada, Shinji Sugahara, Kazuhiro Saito

**Affiliations:** Department of Radiology, Tokyo Medical University Hospital, Tokyo, Japan

**Keywords:** clinical outcomes, dosimetric, isocenter-based prescription, lung cancer, stereotactic body radiotherapy, volume-based prescription, volumetric

## Abstract

**Introduction:**

Stereotactic body radiotherapy (SBRT) has emerged as the standard treatment modality for patients with medically inoperable early-stage non-small cell lung cancer (NSCLC). However, the optimal dose prescription method for SBRT remains controversial, with traditional isocenter-based prescriptions increasingly being replaced by volume-based prescriptions in clinical practice.

**Methods:**

We retrospectively analyzed 88 consecutive patients with T1–T2aN0M0 NSCLC who were treated with SBRT between 2015 and 2024 at our institution. Patients were categorized based on prescription method, and their dosimetric and clinical outcomes were compared.

**Results:**

Dosimetric evaluation results were significantly superior with volume-based prescriptions, demonstrating improved planning target volume (PTV) coverage (95% volume), and higher minimum, mean, and maximum doses (*p* < 0.01 for each). Furthermore, volume-based planning reduced lung radiation exposure, indicated by lower mean lung dose and reduced irradiated lung volumes (>10–40 Gy, *p* < 0.01). Clinically, three-year overall survival and local control rates were markedly higher in the volume-based group than the isocenter-based group (95.0% vs. 64.0%, *p* = 0.01; 93.0% vs. 72.0%, *p* = 0.025). The incidence of Grade 2–5 radiation pneumonitis remained comparable between groups (7.1% vs. 6.5%, *p* = 0.617).

**Conclusion:**

Volume-based SBRT prescriptions enhance both dosimetric quality and clinical efficacy without increasing pulmonary toxicity. Our results highlight volume-based prescription as a preferred strategy for SBRT in patients with early-stage NSCLC.

## 1 Introduction

Stereotactic body radiotherapy (SBRT) represents the current standard of care for early-stage non-small cell lung cancer (NSCLC) in patients deemed medically inoperable or those who decline surgery (1, 2). The technique delivers high local control with minimal toxicity by employing high radiation doses delivered through precise targeting and a limited number of fractions. The success of SBRT has rapidly expanded its clinical application to a wider patient base (3, 4). This rapid adoption has, in turn, generated methodological heterogeneity in treatment protocols across institutions. Such discrepancies include variations in beam energy, number of beams, total dose, and fractionation schemes. A key, unresolved technical variable within these protocols is the specific method utilized for prescribing the SBRT dose.

Historically, SBRT dose was delivered using an isocenter-based prescription (5, 6); however, clinical practice has progressively evolved, with many institutions adopting volume-based prescriptions in recent years (1, 7). Notwithstanding this therapeutic maturity, the optimal prescription technique has yet to be unequivocally determined. Isocenter-based prescription is prone to creating dose heterogeneity that may lead to underdosage at the target margins, while volume-based prescription typically results in a more uniform planning target volume (PTV) dose distribution. A concern with the volume-based approach is its tendency to increase the maximum dose, which could increase the risk of adverse events in adjacent at-risk organs. Due to the limited body of clinical data directly comparing these methods, a clear assessment of their relative merits is warranted. We designed this retrospective study to investigate the dosimetric and clinical performance of isocenter-based versus volume-based SBRT dose prescriptions in patients with early-stage primary lung cancer. Our objectives included quantifying differences in target coverage, radiation exposure to the lung, and the clinical endpoints of overall survival (OS), local control (LC), and incidence of radiation-induced pneumonitis (RP).

## 2 Materials and methods

### 2.1 Ethical approval

The study was approved by the Ethics Review Board of the authors’ institution. All patients provided written informed consent to participate.

### 2.2 Patients characteristics

This retrospective study analyzed a consecutive cohort of patients with T1 or T2a, N0M0 primary lung cancer who were either medically inoperable or declined surgery. All patients received SBRT at the study institution between July 13, 2015, and August 31, 2024. Eligibility required histological confirmation or a highly suspicious nodule on computed tomography (CT) exhibiting malignant ^18^F-fluorodeoxyglucose positron emission tomography (PET) avidity. Patients were excluded if they received concurrent chemotherapy or prior radiation therapy for lung tumors. Only patients with a minimum follow-up duration of 6 months were included (i.e., those followed for less than 6 months or lost to follow-up were excluded). All participants provided written informed consent.

Based on the dose prescription technique employed, patients were stratified into two groups: the isocenter-prescription group (n = 42) and the volume-prescription group (n = 46).

### 2.3 Techniques for simulation and immobilization

Patient immobilization for both the initial simulation and subsequent treatments was achieved using a dedicated body fixation device (HipFix, TOYO MEDIC, Chiyoda, Tokyo, Japan). Radiation treatment planning (RTP) was performed using a four-slice CT scanner (LightSpeed RT 4 slice, GE Healthcare, Mickleton, NJ, United States). To quantify and account for respiratory motion, all patients underwent four-dimensional (4D) CT scanning, during which 2.5 mm slices were acquired synchronously with the respiratory signal. The planned CT scans utilized a slow CT technique, acquiring a single 2.5 mm thickness slice every 4 s. Throughout the simulation and treatment sessions, audio coaching was provided to facilitate a comfortable and consistent breathing rhythm.

### 2.4 Radiotherapy

RTP was performed using the Eclipse treatment planning system (Varian Medical Systems, Palo Alto, CA, United States). SBRT plans were calculated utilizing the Anisotropic Analytical Algorithm (AAA), using a dose calculation grid size of 2.5 mm. The gross tumor volume (GTV), encompassing only the primary tumor, was contoured on the planning CT images. A clinical target volume (CTV) margin was intentionally omitted. The Internal Target Volume (ITV) was determined from the 4D-CT data using the Advantage Workstation (GE Healthcare, Giles, Bucks, United Kingdom). Finally, the PTV value was generated by adding a 5 mm isotropic setup margin to the ITV to account for residual patient setup uncertainties. Lung volume was defined as the total bilateral lung volume excluding the ITV.

**Figure 1:**
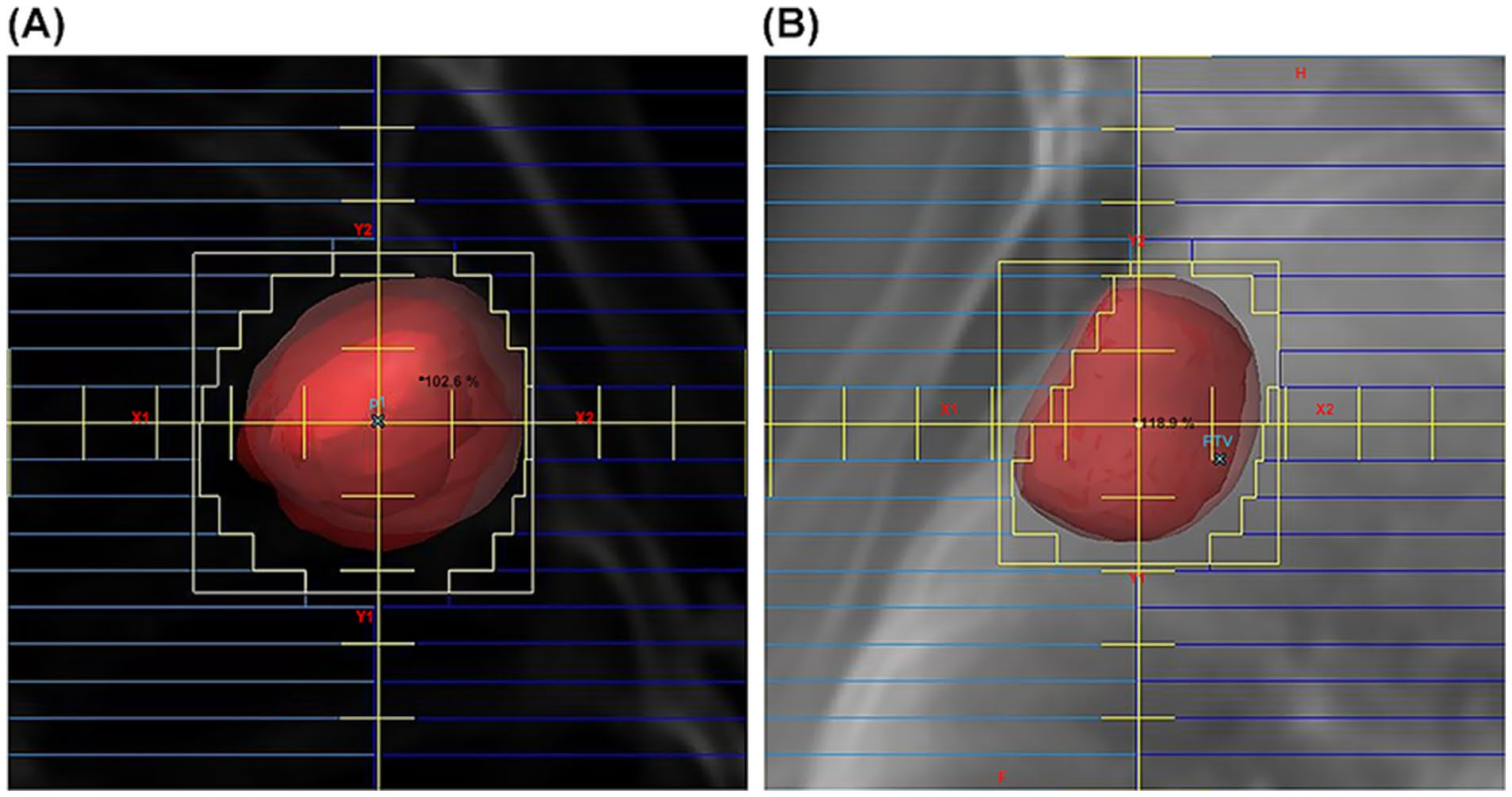
SBRT dose prescription methodologies. The dark red contour represents the CTV, and the light red contour outlines the PTV. The 90% isodose line, relative to the prescribed dose, is shown in orange. **(A)** Isocenter-based prescription field view. The MLC aperture is deliberately opened 5 mm beyond the PTV margin. **(B)** Volume-based prescription field view. The MLC boundary is tightly conformed to the PTV, featuring partial leaf intrusion to ensure a target dose coverage goal of 90% PTV volume.

SBRT was administered using non-coplanar static beams generated by a linear accelerator (CLINAC iX, Varian Medical Systems) operating at 10 MV. Patient setup verification was performed prior to each daily treatment fraction using image guidance via kilovoltage X-ray imaging (On-Board Imager, OBI) and cone-beam CT. The two prescription methods were implemented as follows. In the isocenter-prescription group, the multi-leaf collimator (MLC) was positioned 5 mm beyond the PTV margin, and the full prescribed dose was delivered to the isocenter. In the volume-prescription group, the MLC was adjusted to ensure that 90% of the PTV received the prescribed dose, while the maximum dose was limited to ≤120% of the prescription dose. The typical beam eye view for a field is presented in Figure 1. The standard prescribed dose was 48 Gy delivered in four fractions over 4 days. The dose and number of fractions were modified only for tumors in proximity to high-risk organs (e.g., main trachea, spinal cord, or esophagus) or for exceptionally large tumors. Dose constraints for the pulmonary parenchyma were based on the Japan Clinical Oncology Group (JCOG0403) study protocol (5): a percentage of total lung volume receiving ≥20 Gy (V20) < 20%, a mean lung dose (MLD) < 18 Gy, and V15 < 25%. Fractionation schedules were determined based on tumor location and proximity to organs at risk (OARs). For centrally located tumors or lesions adjacent to critical OARs, more fractionated regimens were generally selected to enhance normal tissue protection. Conversely, shorter hypofractionated regimens were typically employed for peripherally located tumors without close proximity to critical structures.

#### 2.4.1 Dosimetric and clinical assessment

For dosimetric evaluation, the dose covering 95% of the PTV value, denoted as D_95%_, along with the maximum, minimum, and mean doses to the PTV, were calculated and expressed as a percentage of the prescribed dose. The maximum and minimum doses were defined as the absolute voxel-based highest and lowest values within the target volume, respectively. Radiation exposure to the underlying lung was quantified by calculating MLD and the percentage of total lung volume receiving ≥5, 10, 20, 30, and 40 Gy (V5Gy, V10Gy, V20Gy, V30Gy, and V40Gy, respectively). For clinical evaluation, OS, LC, and the incidence of RP were compared between isocenter- and volume-prescription groups. The OS period was defined as the time from the SBRT start date to death from any cause. The LC period spanned from the SBRT start date to the date of local progression or relapse, defined as tumor progression within the irradiated field.

Tumor progression was defined as progressive disease at the primary tumor site, characterized by at least a 20% increase in the longest diameter of the treated lesion compared with the smallest recorded value (nadir), with an absolute increase of ≥5 mm. Recurrence was also considered when increased metabolic uptake suggestive of viable tumor was observed within the treated lesion on PET imaging. RP was graded using the Common Terminology Criteria for Adverse Events version 4.0 (CTCAE v. 4.0).

### 2.5 Patient follow-up

Post-SBRT surveillance involved CT scans performed every 3 months for the first 2 years, followed by semiannual CT scans thereafter. Brain magnetic resonance imaging (MRI) was performed routinely at 3 months post-SBRT and subsequently only when clinical signs or symptoms suggestive of brain involvement were present. Tumor recurrence was confirmed by biopsy in all cases where metastatic disease was not unequivocally apparent on standard CT or PET/CT imaging.

### 2.6 Statistical analysis

Statistical analyses were performed using IBM SPSS Statistics 20.0 (SPSS, Armonk, NY, United States). Baseline characteristics between the two groups were formally compared using Fisher’s exact test for categorical variables and the Mann–Whitney U test for continuous variables. Quantitative data were presented as mean and standard deviation (SD) for normally distributed variables (parametric) or as median and range for nonparametric variables. Qualitative variables were summarized using frequencies and percentages. The Mann–Whitney *U* test was employed to compare the two prescription groups regarding the PTV dose metrics (D_95%_, minimum, maximum, and mean doses) and the lung dose-volume metrics (V5Gy, V10Gy, V20Gy, V30Gy, V40Gy, and MLD). OS and LC were estimated using the Kaplan–Meier method and compared using the log-rank and Cox proportional tests. The incidence of Grade 2–5 RP was compared between groups using Fisher’s exact test. A two-sided *p*-value of <0.05 was considered statistically significant.

## 3 Results

The present study initially included 96 patients with early-stage primary lung cancer. Eight patients were excluded due to an insufficient follow-up period (less than 6 months) or being lost to follow-up, resulting in a final cohort of 88 patients. Patient demographic and clinical characteristics are summarized in Table 1. The isocenter-prescription group (*n* = 42) consisted of 34 men and 8 women, and the volume-prescription group (*n* = 46) included 31 men and 15 women. The median age was comparable between the groups: 79 years (range, 63–90 years) in the isocenter group and 80 years (range, 63–90 years) in the volume group. Median tumor size was 21 mm (range, 10–42 mm) and 21 mm (range, 10–47 mm), respectively. The median follow-up period was 26 months (range, 5–90 months) for the isocenter group and 25 months (range, 8–48 months) for the volume group. The majority of patients in both cohorts had an Eastern Cooperative Oncology Group performance status of 0 or 1. Among the baseline variables, performance status (PS) differed significantly between groups (*p* = 0.002), whereas sex, age, tumor size, and follow-up duration showed no significant differences.

**Table 1:** Characteristics of the patient.

	Prescription		
	Isocenter-based (*n* = 42)	Volume-based (*n* = 46)	*p*
Sex (Male: Female)	34:8	31:15	0.224
Age (median)	63–90 (80)	62–92 (80)	
Age <80: ≥1	22:20	21:25	0.670
PS 0:1:2	24:11:7	40:5:1	
PS 0: ≥1	24:18	40:6	<0.01
Tumor size [median (IQR)]	21.5 mm (16.0–27.0 mm)	22.0 mm (18.0–26.0 mm)	0.953
Total dose/Fraction (*n*)			
48Gy/4Fr	0 (0)	23 (50)	
50Gy/5Fr	14 (33)	9 (20)	
60Gy/10Fr	28 (67)	14 (30)	
Follow-up (median)	5–90 m (26 m)	8–48 m (25 m)	0.124

IQR, Interquartile Range; Fr, Fraction.

### 3.1 Dosimetric results

Dosimetric comparisons for PTV and lung are presented in Tables 2, 3, respectively. The volume-prescription method resulted in significantly superior PTV coverage and dose delivery, with the D_95%_, maximum, minimum, and mean doses all being significantly higher than in the isocenter-prescription group (all *p* < 0.01). Conversely, the volume-prescription method achieved significantly better lung sparing. Specifically, the percentage of lung volume receiving 10, 20, 30, and 40 Gy (V10Gy, V20Gy, V30Gy, and V40Gy) and the MLD were all significantly lower in the volume-prescription group (all *p* < 0.01), but V5 was not significantly different between groups (*p* = 0.121).

**Table 2:** Comparison of dose for PTV.

	Prescription		*p*
	Isocenter-based (*n* = 42)	Volume-based (*n* = 46)	
D95% [median (IQR)]	89.5% (88.0–92.0%)	95.0% (93.0–96.0%)	<0.01
Maximum dose [median (IQR)]	105% (102–107%)	112% (110–115%)	<0.01
Minimum dose [median (IQR)]	82.0% (79.0–87.0%)	90.0% (87.0–90.0%)	<0.01
Mean dose [median (IQR)]	97.0% (95.8–99.0%)	102% (100–104%)	<0.01

Dx%, minimum dose received by x% of the volume of PTV; IQR, Interquartile range.

**Table 3:** Comparison of dose for lung.

	Prescription		*p*
	Isocenter-based (*n* = 42)	Volume-based (*n* = 46)	
V5Gy [median (IQR)]	15.3% (12.6–20.2%)	14.0% (11.3–16.8%)	0.12
V10 Gy [median (IQR)]	9.60% (8.00–11.4%)	5.70% (4.60–7.60%)	<0.01
V20 Gy [median (IQR)]	5.00% (3.30–5.80%)	2.20% (1.80–3.50%)	<0.01
V30 Gy [median (IQR)]	2.55% (1.60–3.10%)	1.25% (0.90–2.00%)	<0.01
V40 Gy [median (IQR)]	1.50% (1.00–2.00%)	0.70% (0.50–1.20%)	<0.01
Mean lung dose [median (IQR)]	5.95Gy (5.40–6.90Gy)	4.80Gy (4.20–5.50Gy)	<0.01

Vx Gy, Irradiated underlying lung volumes of more than x Gy; IQR, Interquartile range.

### 3.2 Clinical outcomes

**Figure 2:**
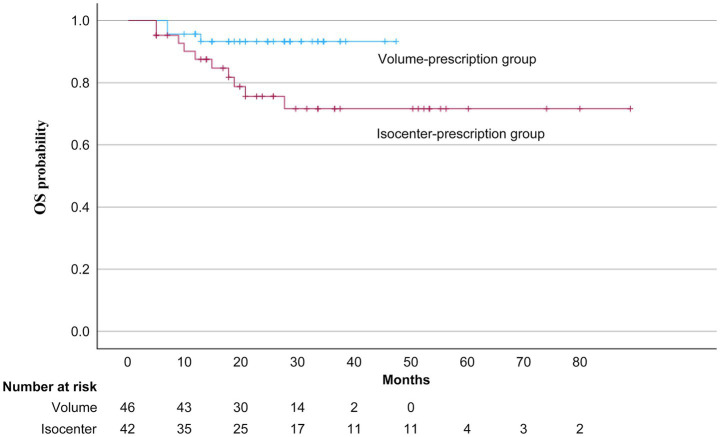
OS probabilities following volume- and isocenter-prescriptions. The volume-prescription group exhibited a significantly higher 3-year OS rate (95.0%) compared to the isocenter-prescription group (64.0%) (*p* = 0.01).

**Figure 3:**
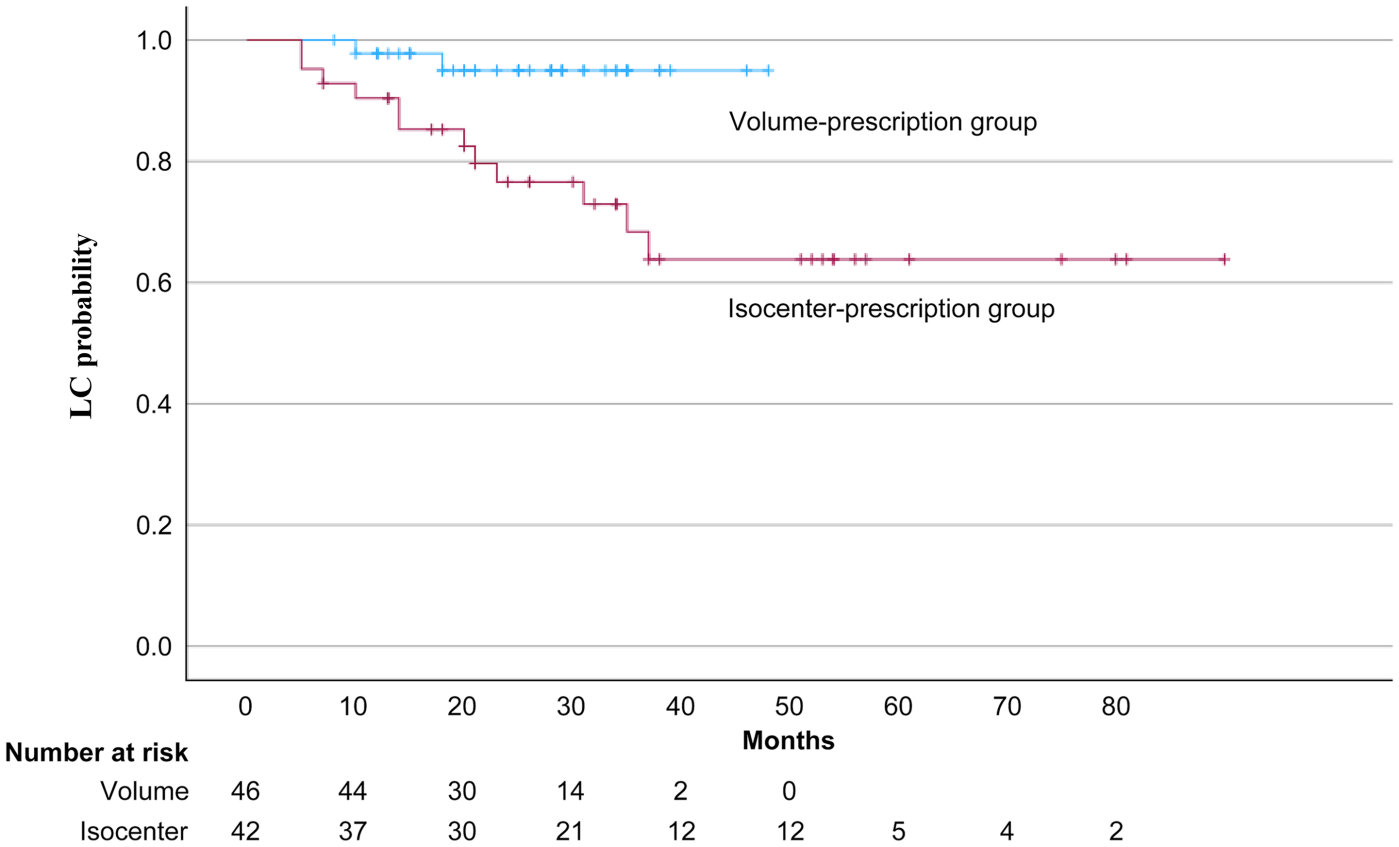
LC probabilities following volume- and isocenter-prescriptions. The volume-prescription group exhibited a significantly higher 3-year LC rate (93.0%) compared to the isocenter-prescription group (72.0%) (*p* = 0.025).

**Figure 4:**
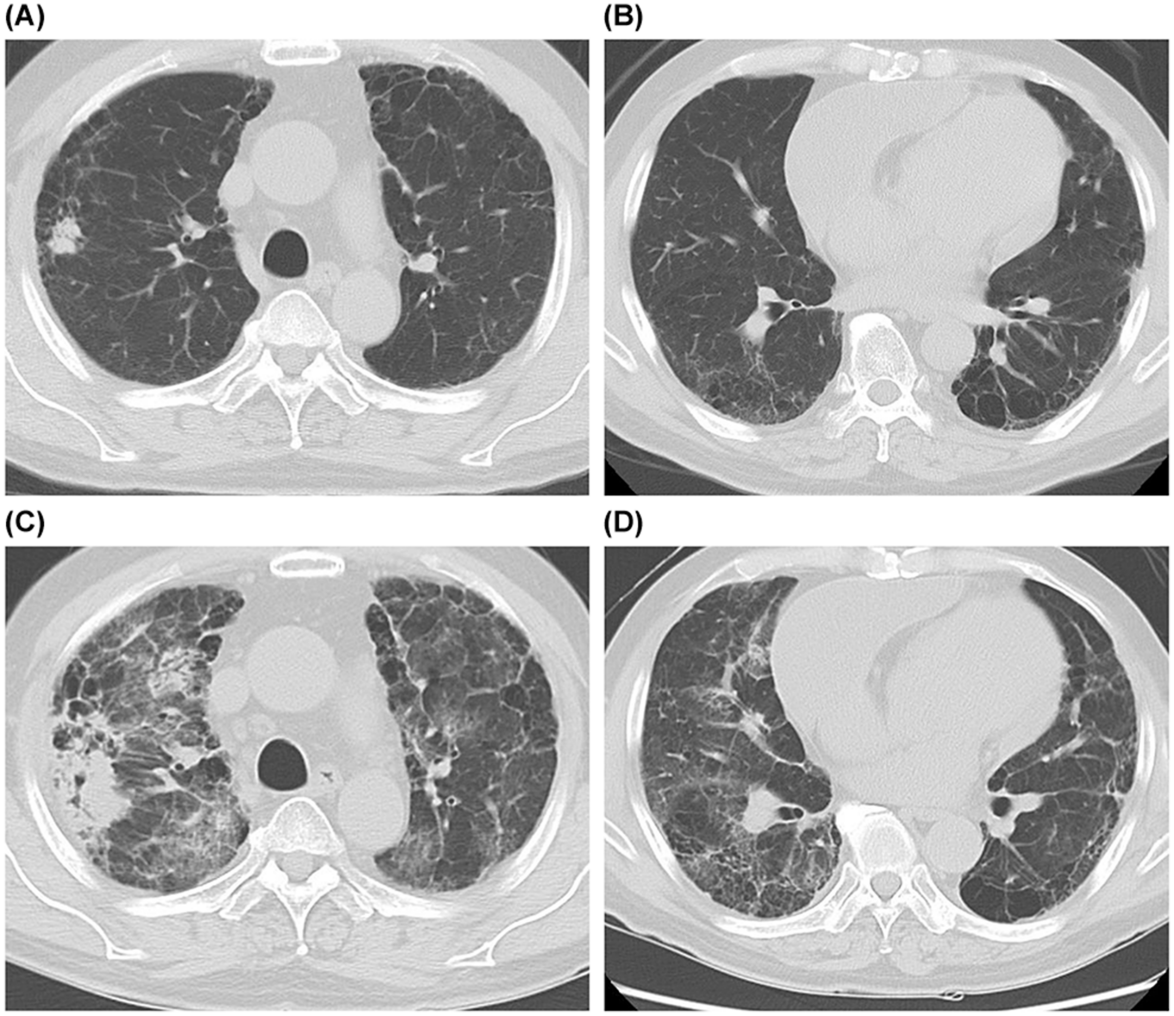
Association between pre-existing interstitial lung disease and fatal RP. **(A,B)** Pretreatment chest CT images showing diffuse interstitial changes in the upper and lower pulmonary fields. **(C,D)** CT images 14 months after SBRT indicating a severe worsening and progression of the interstitial abnormalities, diagnosed clinically as Grade 5 RP.

The three-year OS and LC rates were significantly higher in the volume-prescription group compared to the isocenter-prescription group (Figures 2, 3). Specifically, the 3-year OS probability was 95.0% in the volume group versus 64.0% in the isocenter group (*p* = 0.01). Similarly, the 3-year LC rate was 93.0% in the volume group versus 72.0% in the isocenter group (*p* = 0.025). A multivariable Cox proportional hazards analysis including prescription method and PS was performed. In this model, prescription method remained an independent predictor of OS (HR = 0.197, 95% CI: 0.042–0.930, *p* = 0.04; Table 4). Grade 2–5 RP was observed in 6 patients (6.8%), consisting of five Grade 2 events and one Grade 5 event. The incidence of Grade 2–5 RP was 7.1% (3 patients) in the isocenter-prescription group and 6.5% (3 patients) in the volume-prescription group, demonstrating no statistically significant differences between the two cohorts (*p* = 0.617). The sole Grade 5 RP event occurred in a patient with pre-existing subclinical interstitial lung disease (ILD) (Figure 4).

**Table 4:** Multivariable analysis for overall survival.

Variable	HR	95% CI	*p*
Prescription (Isocenter-based vs. Volume-based)	0.197	0.042–0.930	0.04
PS (0 vs. ≥ 1)	0.659	0.221–1.967	0.45

HR, Hazard ratio; CI, Confidence interval.

## 4 Discussion

This retrospective analysis rigorously compared the dosimetric and clinical performance of isocenter-based and volume-based dose prescription methods in SBRT for early-stage primary lung cancer. Our findings demonstrate that volume-based planning is dosimetrically superior, achieving significantly higher D_95%_, minimum, maximum, and mean doses to the PTV, while concurrently reducing radiation exposure to the surrounding lung parenchyma. This enhanced planning quality translated directly into significantly superior OS and LC for the volume-based group, critically, without increasing the incidence of radiation pneumonitis. Our results underscore the profound clinical relevance of dose prescription methodology, irrespective of the total nominal dose delivered. However, the improved PTV coverage observed in the volume-based prescription group aligns with the inherent definition of the prescription method and its planning objectives. Because target coverage was explicitly prioritized in this approach, higher D95% and near-minimum dose values were anticipated by design. Accordingly, these dosimetric differences should be regarded not as unexpected technical advantages but as reflections of the intrinsic characteristics of the prescription strategy.

Nevertheless, the more homogeneous target coverage achieved with this method may influence intermediate- and high-dose lung exposure, underscoring the need for further evaluation in larger, prospectively controlled studies.

Historically, the majority of SBRT protocols relied on isocenter-based dose prescriptions (4, 8). This conventional approach, however, often leads to inadequate dose coverage at the PTV periphery, thereby elevating the risk of marginal recurrence. In contrast, volume-based prescriptions are designed to ensure that the prescribed dose encompasses the entire PTV, thus optimizing tumor coverage. Our dosimetric data supports this inherent advantage. These findings align with prior radiobiological studies suggesting that higher central or intratumoral biologically effective doses (BEDs)—which account for both total dose and dose per fraction to estimate the true biological impact of radiation—correlate more strongly with LC than peripheral doses (9–11). Specifically, Tateishi et al. highlighted that increased maximum BEDs were associated with improvements in both LC and OS (9), which strongly corresponds with our observation that volume-based prescriptions achieved higher intratumoral doses and superior tumor control. While some reviews, such as that by Eriguchi et al., emphasize the importance of central dose in predicting LC for early-stage NSCLC (12), their analysis did not offer a direct comparison between isocenter- and volume-based prescription methodologies. To our knowledge, studies demonstrating a direct comparison of these two prescription strategies remain limited. The present study represents the first to directly compare the clinical and dosimetric outcomes of these two distinct SBRT prescription strategies, providing novel, high-level evidence supporting the clinical superiority of volume-based prescriptions.

Intriguingly, despite achieving a higher maximum dose within the PTV with the volume-based prescription, the dose-volume metrics for the lung, specifically V10Gy, V20Gy, V30Gy, V40Gy, and the MLD, were significantly lower. Consistent with this improved lung sparing, there was no statistically significant difference in the incidence of RP between the two prescription cohorts. One possible reason for the lack of a measurable difference in RP is the relatively low number of events, which may have limited the statistical power of the analysis. A more compelling consideration is that the development of RP may be primarily influenced by the presence of pre-existing interstitial lung abnormalities, rather than by minor variations in dosimetric parameters. In fact, we previously demonstrated that the presence of interstitial lung abnormalities, rather than lung dose metrics, was the predominant risk factor for this toxicity (13). The observation of the single Grade 5 pneumonitis case in the current study, which occurred in a patient with documented pre-existing ILD, strongly supports this hypothesis.

Our study is subject to several methodological limitations. First, detailed plan quality metrics such as conformity index and gradient index were not systematically recorded in the original dataset and therefore could not be incorporated into the present analysis. Consequently, although reductions in intermediate- and high-dose lung volumes (V10–V40) and MLD were observed in the volume-based prescription group, the extent to which these differences were attributable to variations in conformity or dose gradient cannot be fully determined. Second, its retrospective and single-institutional design introduces inherent selection biases and restricts the generalizability of our findings to other clinical settings. Third, the modest sample size limited the statistical power, particularly for rare events; while the follow-up period was adequate for LC assessment, longer observation is warranted to fully characterize late recurrences and long-term toxicities. Fourth, we did not perform a stratified analysis based on key prognostic factors, such as tumor motion amplitude, central versus peripheral location, or histological subtype, all of which are known to potentially influence treatment response. An additional limitation of this study is the difference in follow-up duration between the two groups. The broader follow-up range observed in the isocenter-based group may reflect era-related variations in treatment implementation. Such temporal differences could have influenced patient selection, treatment techniques, and event accrual. Therefore, potential era effects and unequal follow-up periods should be taken into account when interpreting the clinical outcomes. Finally, although our results clearly support the superiority of the volume-based prescription, the nature of this analysis precluded us from establishing the precise dose–response relationship achieved in large-scale meta-analyses (11, 14–16).

We acknowledge that the sample size of this study is modest and that the median follow-up period (26 months) may limit the assessment of long-term survival. Given the relatively small cohort and the low incidence of toxicity events—particularly radiation pneumonitis—the statistical power to detect differences in rare adverse outcomes was limited. Similarly, the small number of deaths and local recurrences precluded comprehensive multivariable modeling with multiple covariates, as this would have risked overfitting. Nevertheless, because PS differed significantly between groups, a Cox regression analysis adjusting for PS was performed, and prescription method remained independently associated with OS. Larger prospective studies are required to validate these findings.

Another limitation is the imbalance in fractionation schedules and dose distributions between the two groups. Variations in radiobiological dose across treatment regimens may have influenced clinical outcomes—including LC, OS, and toxicity—beyond the effects of prescription strategy alone. Although multivariable analysis was conducted to address baseline imbalances, residual confounding cannot be fully excluded, and the results should therefore be interpreted with caution.

In conclusion, this study unravels novel findings that the SBRT prescription methodology, independent of the total nominal dose, significantly influences both dosimetric parameters and patient clinical outcomes in early-stage lung cancer. The demonstrated superiority of volume-based prescriptions—providing enhanced tumor coverage and improved survival rates without escalating pulmonary toxicity—strongly advocates for its adoption as the standard of care in clinical practice. Future prospective multicenter trials that rigorously adhere to the ICRU 91 reporting standards are essential to definitively validate these findings and further optimize the prescription strategy for lung SBRT.

## Data Availability

The original contributions presented in the study are included in the article/supplementary material, further inquiries can be directed to the corresponding author/s.
